# Comparisons of Dy Utilization Efficiency by DyH_x_ Grain Boundary Addition and Surface Diffusion Methods in Nd-Y-Fe-B Sintered Magnet

**DOI:** 10.3390/ma15175964

**Published:** 2022-08-29

**Authors:** Shuai Guo, Shicong Liao, Xiaodong Fan, Guangfei Ding, Bo Zheng, Renjie Chen, Aru Yan

**Affiliations:** 1 CISRI & NIMTE Joint Innovation Center for Rare Earth Permanent Magnets, Ningbo Institute of Material Technology and Engineering, Chinese Academy of Sciences, Ningbo 315201, China; 2 Key Laboratory of Magnetic Materials and Devices, Ningbo Institute of Material Technology and Engineering, Chinese Academy of Sciences, Ningbo 315201, China; 3 University of Chinese Academy of Sciences, Beijing 100049, China

**Keywords:** Dy utilization efficiency, DyH_x_, addition method, diffusion method, Nd–Y–Fe–B magnet

## Abstract

Using the heavy rare earth Dy element to improve coercivity is the most common solution for hindering the reduction in magnetic properties in the Nd–Fe–B magnet, and the effective utilization of Dy has become the focus of research in industrial society. In this work, we investigated the influence of DyH_x_ addition and diffusion methods on the microstructure, magnetic performance, and thermal stability of the Nd–Y–Fe–B magnet with a Y-rich core structure. The coercivity of the DyH_x_ addition magnet increases from 9.45 kOe to 15.51 kOe when adding 1.6 wt.% DyH_x_, while the DyH_x_ diffusion magnet increases to 15.15 kOe. According to the analysis of the microstructure and elemental distribution, both Dy-rich shells were basically formed due to the diffusion process of Dy atoms. The Dy-rich shell in the DyH_x_ addition magnet was similar with the original core–shell structure in the Nd–Y–Fe–B magnet. However, the distinct dual-shell structure consisting of a thinner Dy-rich shell and a Y-lean shell was constructed in the DyH_x_ diffused magnet, contributing to the superior coercivity increment and Dy utilization efficiency. Furthermore, the remanence of the DyH_x_ diffused magnet is up to 12.90 kG, which is better than that of the DyH_x_ addition magnet (12.59 kG), due to fewer Dy atoms entering the 2:14:1 matrix grain to cause the antiferromagnetic coupling with Fe atoms. Additionally, the thermal stability of the DyH_x_ diffusion magnet is also better than that of the DyH_x_ addition magnet, owing to the elevated coercivity at room temperature, which expands the application range of the Nd–Y–Fe–B magnet to a certain extent.

## 1. Introduction

Recently, the consumption of Nd–Fe–B-type magnets has rapidly increased all over the world because of their outstanding performance in electric motors, hybrid vehicles, wind generators, and electronic communication devices, etc. [1,2,3,4,5,6,7,8,9,10]. This leads to a large consumption of rare earth metal resources, such as Pr, Nd, Dy, or Tb, and a significant increase in the cost of magnets. Simultaneously, large amounts of the rare earth elements, such as Y, which possesses the merits of low cost and high abundance in the Earth’s crust, have been left unused [11,12,13,14]. Thus, the substitution of the rare earth element Y for Nd [15,16,17] has attracted much attention in the permanent magnetic society.

However, the coercivity of the Nd–Y–Fe–B magnet significantly deteriorates after doping with Y due to the lower anisotropy field of Y_2_Fe_14_B (*H*_a_ = 26 kOe) compared with Nd_2_Fe_14_B (*H*_a_ = 73 kOe) [11,12]. The heavy rare earth elements Dy and Tb have been introduced directly to improve the coercivity of Nd–Fe–B magnets for the strong anisotropy fields of Dy_2_Fe_14_B (*H*_a_ = 150 kOe) and Tb_2_Fe_14_B (*H*_a_ = 220 kOe) [4] at room temperature. Generally, a (Dy/Tb, Nd)_2_Fe_14_B phase with enhanced anisotropy field will be formed on the matrix grain surface of the magnet, while Dy/Tb is doped into the magnet as the oxides [18,19], hydrides [20,21,22], fluorides [23,24] and so on, but the coercivity of the magnet is not enhanced as much as expected. Another method to introduce heavy rare earth Dy/Tb is the grain boundary diffusion process (GBDP) which is also the most important inventions in the last two decades for the rare earth (RE) permanent magnets industry [25,26,27]. It has been proposed to enhance the coercivity of Nd–Fe–B magnets using Dy/Tb diffused along the grain boundary into the interior and the matrix grains to construct a Dy/Tb-rich shell [28]. On the other hand, the remanence of Nd–Fe–B magnets will be heavily decreased due to the antiferromagnetic coupling among the Dy/Tb atoms and the Fe atoms [29,30]. Therefore, it is important to introduce heavy rare earth elements without damaging the other magnetic properties, that is to say, how to efficiently utilize heavy rare earth Dy/Tb in small quantities has become the theme of the current research.

In this work, the effect of two different ways of Dy introduction, i.e., DyH_x_ addition and DyH_x_ diffusion methods, on the magnetic properties, microstructures, and thermal stability of the Nd–Y–Fe–B magnet has been studied. The results show that the Dy utilization efficiency of diffusion method is superior to the addition method, results which are significantly meaningful in their application of the science of Nd–Y–Fe–B magnets.

## 2. Materials and Methods

### 2.1. Experimental Procedure

Alloy strips with a nominal composition of (PrNd)_20.66_Y_6.88_B_0.98_M_8.5_Fe_bal_ (wt.%, M = Al, Cu, Co, named as Y25) were prepared by a strip-casting (SC) technique. The alloy strips were crushed into powders with an average particle size of 2.2 μm by hydrogen decrepitation along with N_2_-jet milling. The DyH_x_ powder with an average size of about 1 μm was prepared by hydrogen absorption fragmentating and N_2_-jet milling. Then, 1.6 wt.% DyH_x_ powder was added into the Y25 matrix powder as a modifier. The Y25 and the mixed powders were pressed and oriented under a magnetic field of 2T in a protective nitrogen atmosphere, followed by iso-static compaction under a pressure of 150 MPa. Subsequently, the sintering process was performed at 1060–1080 °C for 2 h in a vacuum atmosphere, followed by a two-step annealing at 900 °C for 2 h and 500 °C for 2 h to obtain the final magnets. In order to fabricate the diffusion matrix, the sintered Y25 magnet was cut into a cylinder with the size of Φ10 mm × 5 mm, and the diffusion source was prepared by mixing the DyH_x_ powder and alcohol under the mass ratio of 1:1. The Y25 cylinder was immersed into the diffusion source for 5 s to obtain a uniform DyH_x_ layer on the surface, then the sample was heat treated at 900 °C for 10 h, followed by annealing at 500 °C for 2 h. The final average Dy content of the diffusion magnet was 0.421 wt.%, obtained by using ICP-OES. For ease of description, the Y25 magnet, the 1.6 wt.% DyH_x_ addition magnet, and the DyH_x_ diffusion at 900 °C magnets were named as the original magnet, the DyH_x_ addition magnet, and the DyH_x_ diffusion magnet, respectively.

### 2.2. Characterization and Analysis Methods

The room-temperature and elevated-temperature magnetic properties of the magnets were measured using a magnetic measurement system (NIM-500C). The microstructure observation of the samples was performed in the back scattered mode of scanning electron microscopy (SEM, FEI QUANTA 250, FEI Company, Hillsboro, OR, USA). The contents of elements in the magnets were obtained by using energy-dispersive X-ray spectroscopy (EDS). The elemental concentration mapping was conducted using an electron probe microanalyzer (EPMA, JEOL JXA-8100, Tokyo, Japan). The contents of rare earths in the magnets were examined using a glow discharge emission spectrum (GD-OES, Spectruma Analytik GMBH 750HP, Hof, Germany). The irreversible loss of the magnetic flow was investigated with a Helmholtz coil after exposing the samples at elevated temperatures for 0.5 h in open circuit.

## 3. Results and Discussion

### 3.1. Magnetic Properties

**Figure 1:**
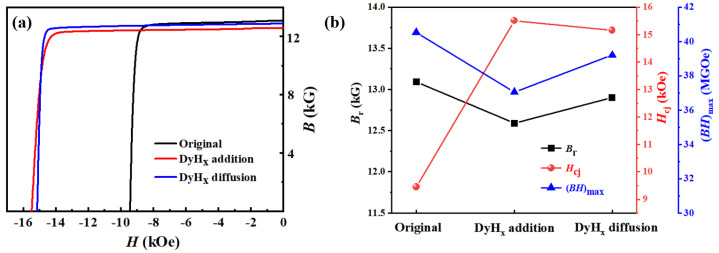
(**a**) The demagnetization curves of the original magnet, the DyH_x_ addition magnet, and the DyH_x_ diffusion magnet; (**b**) the variation curves of the corresponding *B*_r_, *H*_cj_, and (*BH*)_max_ of the three types of magnets derived from (**a**).

Figure 1a demonstrates the demagnetization curves of the original magnet, the DyH_x_ addition magnet, and the DyH_x_ diffusion magnet. The variation curves of the corresponding *B*_r_, *H*_cj_, and (*BH*)_max_ of the three type magnets are also shown in Figure 1b. The room temperature magnetic properties of the magnets are listed in Table 1. The results show that the coercivity of the addition magnet is 15.51 kOe, which is a slightly higher than that of the diffusion magnet (15.15 kOe), and the coercivities of the two DyH_x_ treated magnets are much higher than that of the original magnet (9.45 kOe). Unfortunately, the remanences of the addition magnet and the diffusion magnet, respectively, reduce to 12.59 kG and 12.90 kG compared with the original magnet (13.09 kG). Furthermore, the average Dy content in the diffusion magnet, obtained by using ICP-OES, is 0.421 wt.%, which is much lower than that of the addition magnet. In this work, the utilization efficiency of Dy can be defined as the ratio of Δ*H*_cj_ to the average Dy content in the magnet. Thus, the coercivity increment of the diffusion magnet is significantly improved to 13.5 kOe/(wt.% Dy), which is much higher than that of the addition magnet (about 3.8 kOe/(wt.% Dy)). The previous studies [29,30] indicate that the remanence deteriorates after Dy doping into the Nd–Fe–B magnet due to the anti-ferromagnetic coupling effect between the heavy rare earth Dy and the transition metal Fe. Thus, the DyH_x_ diffusion magnet has a higher remanence compared to the DyH_x_ addition magnet. These changes in coercivity and remanence eventually lead to variety in the maximum energy product (*BH*)_max_. Although the maximum energy products of the addition and diffusion magnets are decreased compared with the original magnet, the (*BH*)_max_ of the diffusion magnet is much higher than that of the addition magnet. The (*BH*)_max_ of the diffusion magnet decreased by only 3.2% compared with the original magnet, which shows significant merit for the Y-containing magnet.

### 3.2. Microstructure and Elemental Distribution

**Figure 2:**
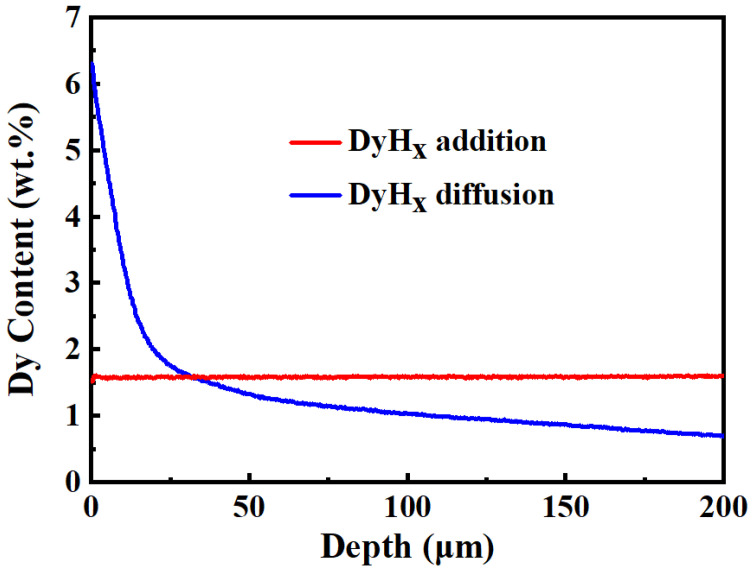
The Dy concentration distributions of the DyH_x_ addition and diffusion magnets in the depth range of 0–200 μm.

Figure 2 shows the distribution of Dy element in the DyH_x_ addition, and diffusion magnets from the surface to an interior of 200 μm depth. The Dy distributes uniformly in the DyH_x_ addition magnet, and the Dy content is about 1.6 wt.% in the whole magnet. In the DyH_x_ diffusion magnet, Dy concentration is inhomogeneous and decreases with the increasing diffusion depth. From the surface to the interior about 30 μm of the magnet, Dy concentration dramatically drops with the increasing depth, but it is much higher than that of the addition magnet. At 30 μm depth from the surface of the magnets, the Dy element concentration of the diffusion magnet starts to be lower than that of the addition magnet, and their difference becomes larger with the increasing depth. It can be considered that the utilization efficiency of heavy rare earth Dy by the grain boundary diffusion method is much higher than that of dual alloy method with the same coercivity enhancement.

**Figure 3:**
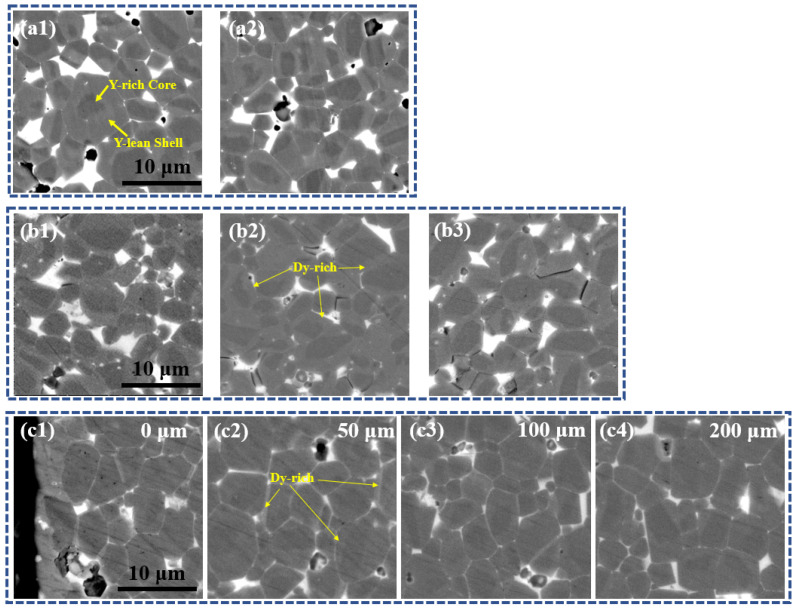
The microstructures of the original magnet, DyH_x_ addition magnet, and the DyH_x_ diffusion magnet. (**a1**,**a2**) are two different positions of the original magnet, (**b1**–**b3**) are three random positions in the DyH_x_ addition magnet, and (**c1**–**c4**) are the microstructures at the depths of 0 μm, 50 μm, 100 μm, and 200 μm from the surface of the DyH_x_ diffusion magnet, respectively.

The microstructures of the DyH_x_ addition magnet and the DyH_x_ diffusion magnet have also been obtained by using SEM, and are shown in Figure 3. Figure 3(a1,a2) are two different positions of the original magnet, which are shown that the core–shell structure with Y-rich core and Y-lean shell was formed during the heat treatment processes. Figure 3(b1–b3) represent the microstructures of three random positions in the DyH_x_ addition magnet, which clearly demonstrates that the microstructure inside the addition magnet remains basically the same. Additionally, the Dy-rich shells have been formed in the outer layer of the matrix grains. Figure 3(c1–c4) are the microstructures at the depths of 0 μm, 50 μm, 100 μm, and 200 μm from the surface of the DyH_x_ diffusion magnet. The results show that the microstructure of the magnet gradually varies from the surface to the interior. The Dy-rich shells are also formed in the outer layer of the matrix grains of the DyH_x_ diffusion magnet, while the thickness of the Dy-rich shell gradually decreases from the surface to the interior. Furthermore, the thickness of the Dy-rich shell in the DyH_x_ diffusion magnet is far less than that of the DyH_x_ addition magnet, which means that the DyH_x_ grain boundary diffusion method consumes much lower heavy rare earth Dy compared to the dual alloy doping method. On the other hand, the core–shell structure of the DyH_x_ addition magnet is similar to the original magnet, except that the shell contains the Dy element, while a double shell structure was formed in the DyH_x_ diffusion magnet that will be discussed below. Moreover, it is also obvious that the grain boundary phase of the DyH_x_ diffusion magnet is clearer and more continuous than that of the DyH_x_ addition magnet, which also contributes to the improvement of coercivity.

**Figure 4:**
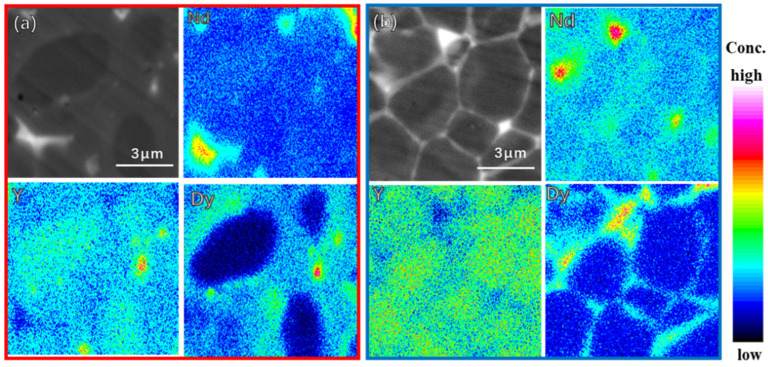
EPMA elemental mappings of the DyH_x_ addition magnet (**a**) and the DyH_x_ diffusion magnet (**b**).

The distribution of Dy in the magnet plays a significant role in the improvement of magnetic properties and therefore, the detailed elemental distributions of the addition and diffusion magnets was investigated, as shown in Figure 4. The results show that, in the DyH_x_ addition magnet, large amount Dy atoms diffuse into the matrix grains to form (Nd, Dy)_2_Fe_14_B shells, which can greatly enhance the surface magnetocrystalline anisotropy field of the matrix grains to improve the coercivity. However, there are also large numbers of Dy atoms agglomerating in the triple junction area, which is unfavorable to the improvement of coercivity. In the DyH_x_ diffusion magnet, Dy atoms diffuse through grain boundaries into the interior of the magnet. On one hand, Dy atoms diffusion into the surface of the matrix grains to form a thinner (Nd, Dy)_2_Fe_14_B layer and, thus, fewer heavy rare earth atoms enter the matrix grain to decrease the saturation magnetization of the main phase, leading to the higher remanence of the final diffusion magnet. On the other hand, partial Dy atoms infiltrate into the deeper part inside the magnet along the grain boundary to broaden the thickness of thin grain boundary, resulting in a stronger magnetic isolation effect between the adjacent matrix grains and higher coercivity for the final diffusion magnet.

**Figure 5:**
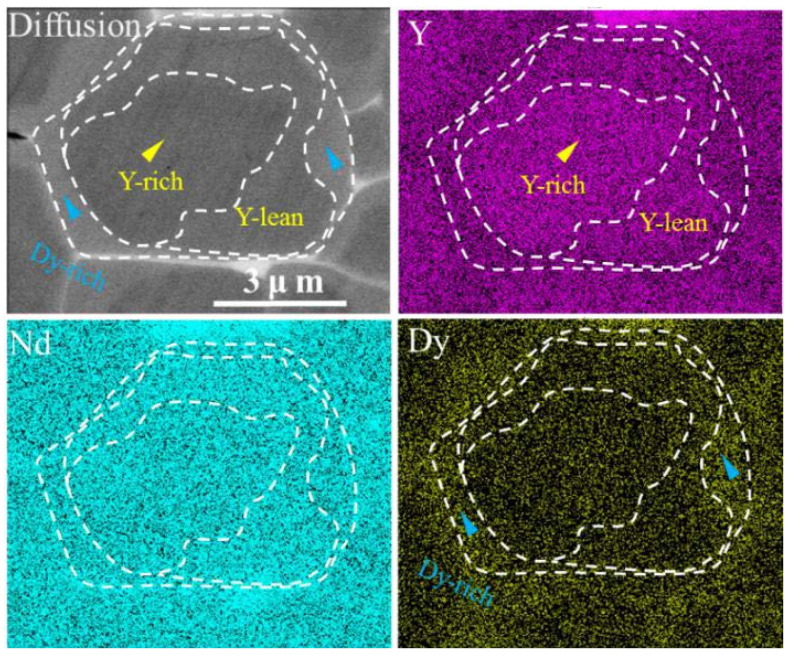
Back-scattered SEM image mappings for the DyH_x_ diffusion magnet at the depth of 50 μm.

Figure 5 shows the elemental distribution of Dy in the matrix grain at a depth of 50 μm for the DyH_x_ diffusion magnet. It can be seen that three layers with different contrasts appear in the matrix grains after the grain boundary diffusion process. In addition to the core–shell structure where Y forms a Y-rich core and a Y-lean shell in the interior of the matrix grain, Dy also diffuses into the outside of the Y-lean shell to form a Dy-rich shell. The Dy-rich shell is brighter in SEM backscatter image due to the larger atomic weight of Dy than those of Nd and Y. From the center to the surface, the matrix grain forms a double-shell structure with a Y-rich core, a Y-lean Dy-lean shell as well as a Y-lean Dy-rich shell in which the anisotropy field is sequentially enhanced. Therefore, the anisotropy field of the matrix grain is improved after the diffusion process and, thus, the coercivity has been increased.

### 3.3. Thermal Stability

**Figure 6:**
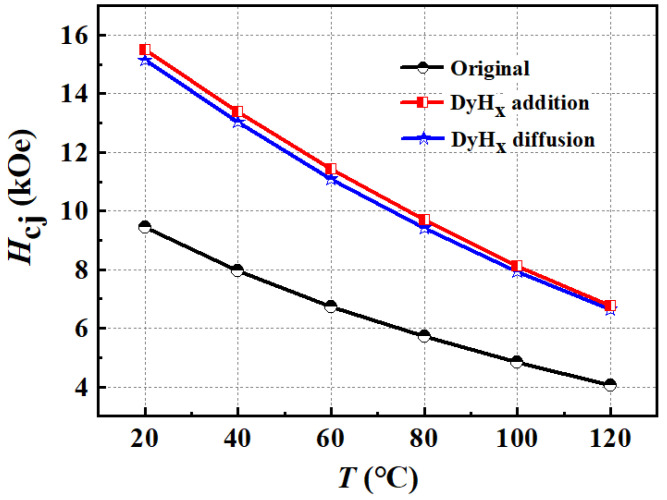
The coercivity versus temperature of the original magnet, the DyH_x_ addition magnet, and the DyH_x_ diffusion magnet.

When Nd–Fe–B magnets are used in motors and electronic products, they always need to be able to perform at high temperature due to the heating of the devices, so the magnetic performances of the magnet at high temperatures are very important performance indicators. The temperature stability of Nd–Fe–B magnet is closely related to their microstructures and intrinsic properties. In general, high coercivity is beneficial for magnet temperature stability. Therefore, the dependences of coercivity on the temperature of the original magnet, the DyH_x_ addition magnet, and the DyH_x_ diffusion magnet have been obtained, as shown in Figure 6. The temperature coefficients of the coercivity from 20 °C to 120 °C have also been calculated using the formula $\beta =\frac{H_{T}-H_{T_{0}}}{H_{T_{0}}\left(T-T_{0}\right)}*100\%$, where *β* is the coercivity temperature coefficient from *T*_0_ to *T*, which is shown in Table 2.

Figure 6 shows that the coercivities of the magnets decrease with the increasing temperature. While at the same temperature, the coercivities of the DyH_x_-treated magnets are much higher than that of the original magnet. In the range of 20–120 °C, the coercivity temperature coefficient *β* of the original magnet is −0.5968%/°C. After introducing DyH_x_, the coercivity temperature stability has been improved significantly. The *β* of the DyH_x_ addition and diffusion magnets are, respectively. −0.5632%/°C and −0.5614%/°C, which means that the coercivity temperature stability of the DyH_x_ diffusion magnet is slightly better than that of the DyH_x_ addition magnet.

**Figure 7:**
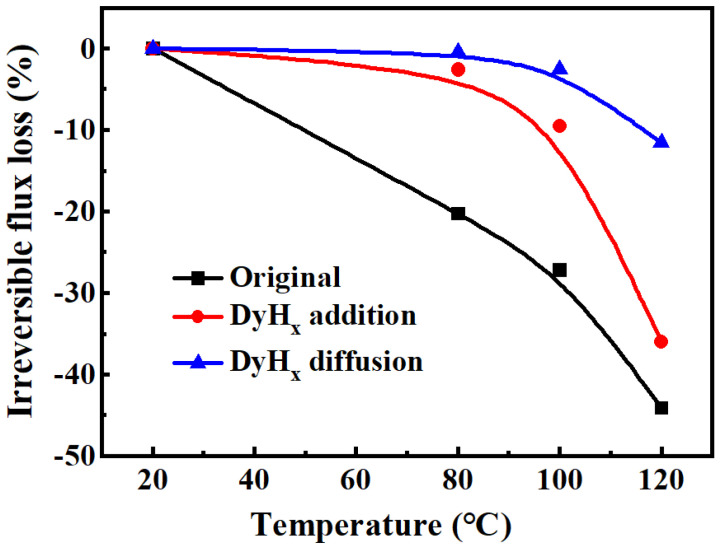
Irreversible loss of flux versus temperature of the original magnet, the DyH_x_ addition magnet, and the DyH_x_ diffusion magnet.

The irreversible loss of the magnetic flux is also an important parameter to evaluate the high temperature performance of the magnet. As the temperature increases, the magnetic flux will also decrease, but the magnetic flux will recover after the temperature drops to room temperature, thus, the irreversible flux part is called the irreversible magnetic flux loss. Moreover, the smaller the irreversible magnetic flux loss of the magnet, the better the high temperature resistance of the magnet. Figure 7 shows the irreversible flux loss versus aging temperature for the initial magnet, the DyH_x_ addition magnet, and the DyH_x_ diffusion magnet. The results show that the irreversible flux loss of the initial magnet is about 20% after being treated at 80 °C for 2 h. However, the irreversible flux losses of the DyH_x_-treated magnets significantly reduce, which means that the loss values of DyH_x_ addition and diffusion magnet are 2.6% and 0.43%, respectively. Moreover, the irreversible flux loss of the DyH_x_ addition magnet is about 10% when the temperature increases to 100 °C, but the same loss value has been obtained in the DyH_x_ diffusion magnet at higher that 120 °C, indicating the enhanced high temperature stability of the DyH_x_ diffusion treatment compared to DyH_x_ addition.

### 3.4. Discussions

The above investigations show that the magnetic performances and the high temperature stability of the DyH_x_ diffusion magnet is higher than that of the DyH_x_ addition magnet, which is mainly attributed to the differences of microstructures and elemental distributions due to the way that Dy enters the magnets. When they are used as a grain boundary additive, the Dy atoms generate liquid phases to fill the gaps between grains and aggregate in the matrix phase grain intersection areas to form triple junction phases during the sintering process. While they are treated as diffusion sources, the Dy atoms diffuse through the melted grain boundary phase during the diffusion process and do not accumulate at the grain boundary. At the same time, the melting Dy repairs the defects among the matrix grain and the grain boundary phase, and the distance between the matrix grain is also broadened.

The Dy-rich shells which are formed in the matrix grains of DyH_x_ addition and diffusion magnets are also significantly different. The thickness of the (Nd, Dy)_2_Fe_14_B shell in the addition magnet is higher than that of diffusion magnet, which is mainly due to the fact that the Dy atom at the grain boundary is more likely to enter the matrix grains and be substituted by an Nd atom at elevated temperatures. In the grain boundary diffusion process, a thin Dy-rich shell is formed in the surface of the matrix grain, which greatly improves the utilization of the Dy element. Moreover, it also prevents too much Dy from entering the matrix grains to reduce the saturation magnetization. Therefore, the grain boundary diffusion method of Dy has a higher utilization efficiency in improving the coercivity.

## 4. Conclusions

We investigated the effect of DyH_x_ addition and diffusion methods on the microstructure, magnetic performance, and thermal stability of the Nd–Y-Fe–B magnet. The coercivity of the original magnet increased from 9.45 kOe to 15.51 kOe for the DyH_x_ addition magnet and 15.15 kOe for the DyH_x_ diffusion magnet. However, the coercivity increment of the Dy element of the diffusion method was up to 13.5 kOe/(wt.% Dy), much higher than the addition method (about 3.8 kOe/(wt.% Dy)). Moreover, the remanence of the DyH_x_ diffusion magnet was as high att 12.90 kG, which was better than the DyH_x_ addition magnet (12.59 kG). These superior magnetic performances of DyH_x_ diffusion magnet are mainly due to the outstanding utilization efficiency of Dy that diffused into the outer layer of the matrix grain to form a thinner Dy-rich shell and infiltrated along the grain boundary to construct a clear and continuous grain boundary phase. The Dy-rich shell in the DyH_x_ addition magnet was similar, with the original core–shell structure in the Nd–Y–Fe–B magnet. However, the distinct dual-shell structure consisting of a thinner Dy-rich shell and Y-lean shell was constructed in the DyH_x_ diffused magnet, contributing to the superior coercivity increment and Dy utilization efficiency. Based on the distribution characteristic of Dy in the magnet, the thermal stability of the DyH_x_ diffusion magnet is also superior to the DyH_x_ addition magnet, which will greatly expand the application range of the Nd–Y–Fe–B magnet.

## Figures and Tables

**Table 1:** The room temperature magnetic properties of the magnets.

Sample	*B*_r_ (kG)	*H*_cj_ (kOe)	(*BH*)_max_ (MGOe)
Original	13.09	9.45	40.52
DyH_x_ addition	12.59	15.51	37.05
DyH_x_ diffusion	12.90	15.15	39.20

**Table 2:** The coercivity temperature coefficient *β* from 20 °C to 120 °C of the magnets.

Magnets	*β* (%/°C)
Original	−0.5968
DyH_x_ addition	−0.5632
DyH_x_ diffusion	−0.5614

## Data Availability

The data presented in this study are available on request from the corresponding author.
